# Supplementary material for: Human serum triggers antibiotic tolerance in Staphylococcus aureus
Source: Nat Commun. 2022 Apr 19;13:2041. doi: 10.1038/s41467-022-29717-3 (PMC9018823; doi:10.1038/s41467-022-29717-3)
Supplement: Supplementary file 1 — Supplementary information [file 41467_2022_29717_MOESM1_ESM.pdf]

Human serum triggers antibiotic tolerance in *Staphylococcus aureus*

Elizabeth V. K. Ledger[1], Stéphane Mesnage[2] and Andrew M. Edwards[1*]

Supplementary information

Supplementary Figures 1-15

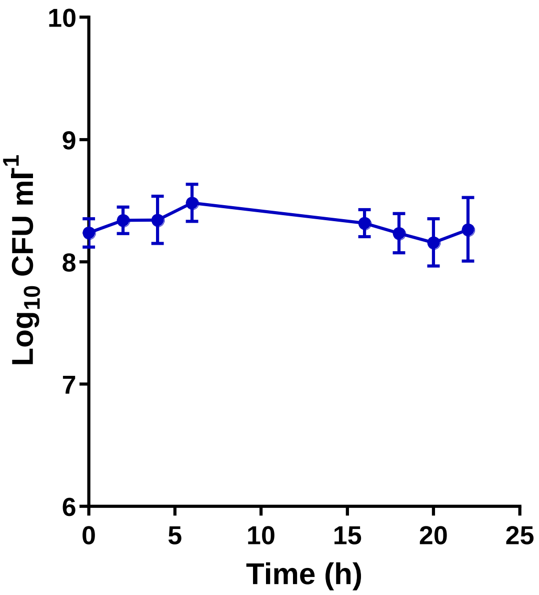

Supplementary Fig. 1. *S. aureus* CFU counts do not change during incubation in human serum. Log_{10} CFU ml^{-1} during incubation of TSB-grown *S. aureus* in human serum for 22 h. Graph represents the geometric mean ± geometric standard deviation of three independent experiments. Data were analysed by one-way ANOVA and no statistically significant differences were observed (log_{10} CFU ml^{-1} at time points vs at 0 h). Source data are provided as a Source Data file.

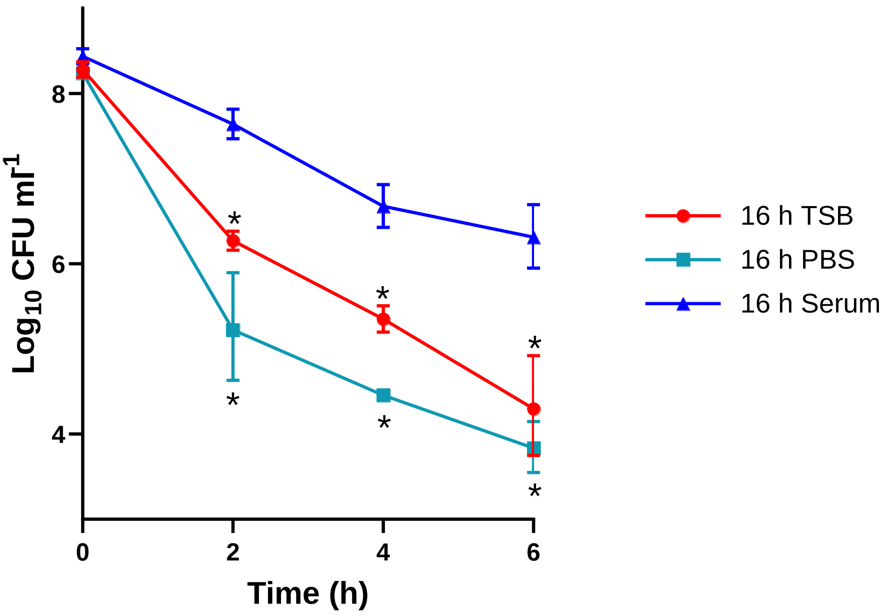

Supplementary Fig. 2. Incubation of *S. aureus* in serum but not TSB or PBS for 16 h results in tolerance towards daptomycin. TSB-grown *S. aureus* cultures were incubated for 16 h in TSB, PBS or serum, adjusted to 2 x 10^8 CFU ml^{-1} where necessary and resuspended in fresh serum containing 80 µg ml^{-1} daptomycin. Log_{10} CFU ml^{-1} was measured over 6 h. Graph represents the geometric mean ± geometric standard deviation of three independent repeats and data were analysed by two-way ANOVA with Tukey's *post-hoc* test (serum-adapted vs PBS/TSB-adapted at each time point). * P = 0.0016 (TSB 2 h), 0.0065 (TSB 4 h), 0.022 (TSB 6 h), 0.0292 (PBS 2 h), 0.0075 (PBS 4 h), 0.0023 (PBS 6 h). Source data are provided as a Source Data file.

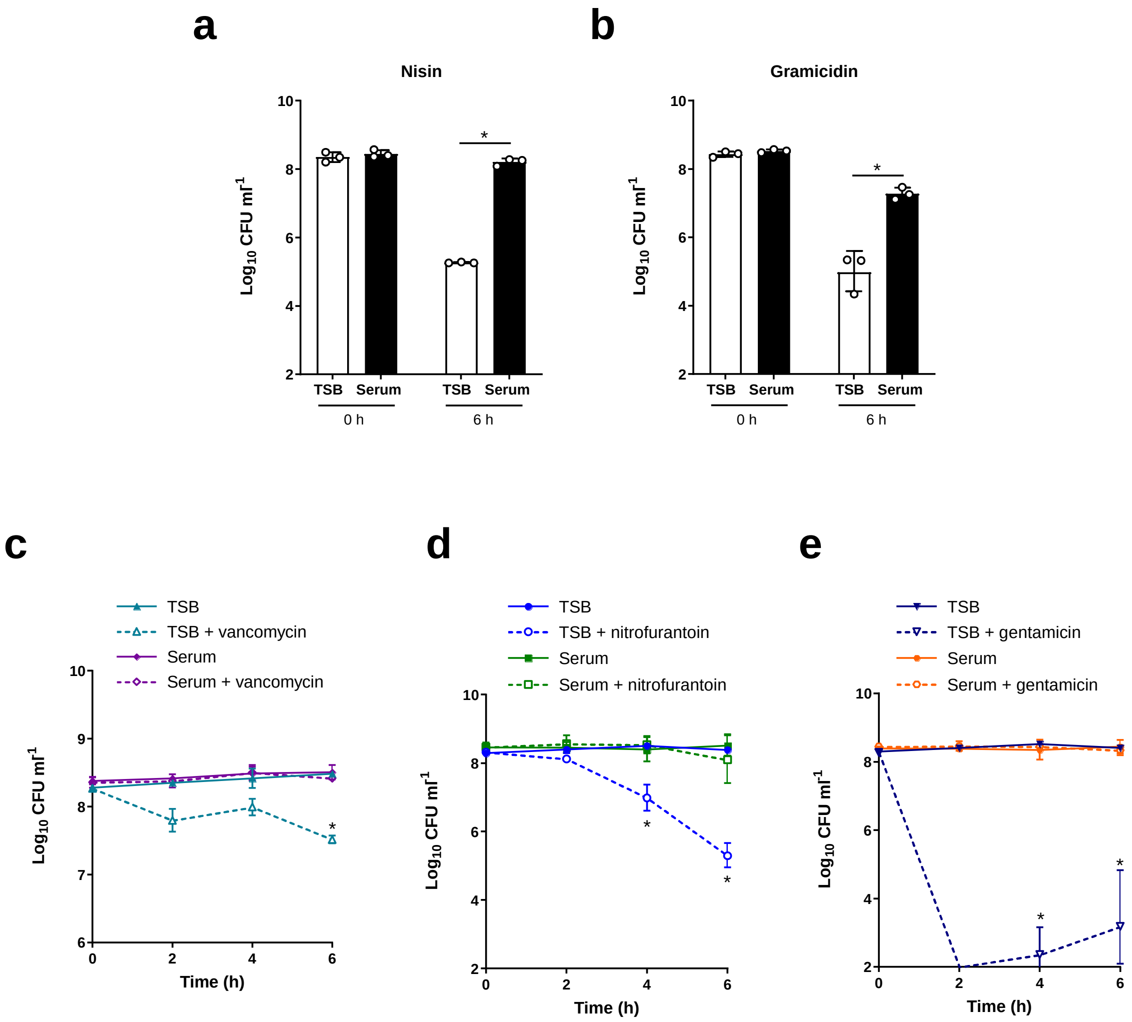

Supplementary Fig. 3. Incubation of *S. aureus* in serum results in tolerance towards antimicrobials from various classes. Log_{10} CFU ml^{-1} of TSB-grown and serum-adapted cultures of *S. aureus* USA300 WT after/throughout a 6 h incubation in serum with 128 µg ml^{-1} nisin (**a**), 160 µg ml^{-1} gramicidin (**b**), 160 µg ml^{-1} vancomycin (**c**), 160 µg ml^{-1} nitrofurantoin (**d**) or 40 µg ml^{-1} gentamicin (**e**). Graphs represent the geometric mean ± geometric standard deviation of three (**a, b, d, e**) or two (**c**) independent replicates. Graphs in **a** – **b** were analysed by two-way ANOVA with Sidak's *post-hoc test* (* for **a**, $P < 0.0001$. For **b**, $P < 0.0001$. Graphs in **c** – **e** were analysed by two-way ANOVA with Tukey's *post-hoc* test; TSB-grown compared to serum-adapted at each time-point. (* for **c**, $P = 0.0103$. For **d**, $P = 0.0197$ (4 h), 0.0257 (6 h). For **e**, $P = 0.0122$ (4 h), 0.0491 (6h)). In **e**, some data points fell below the limit of detection of 100 CFU ml^{-1}. Source data are provided as a Source Data file.

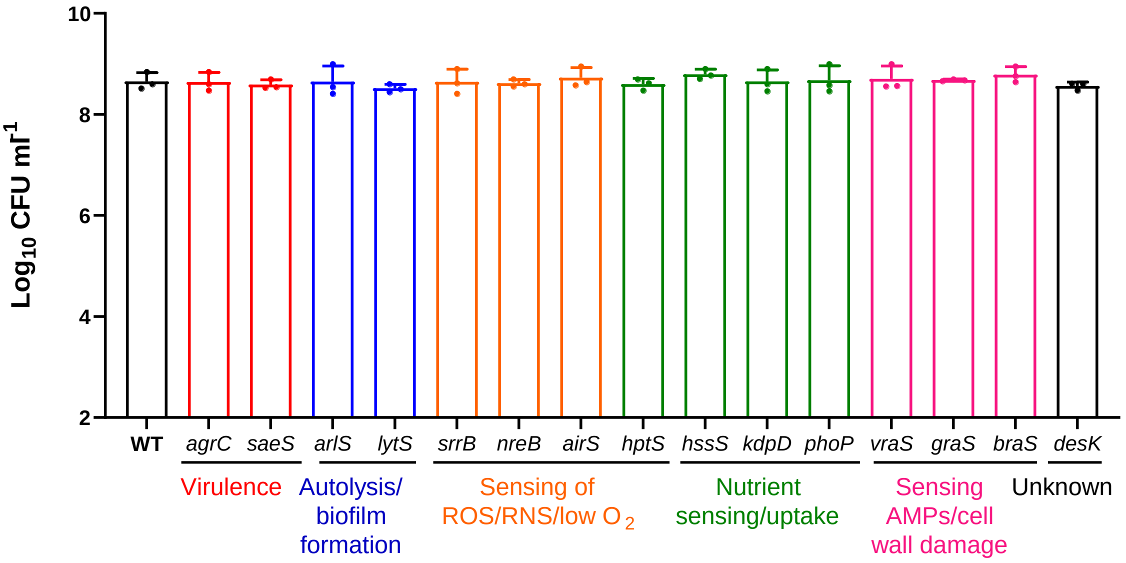

Supplementary Fig. 4. TCS-deficient mutants survive as well as WT in human serum. Log$_{10}$ CFU ml^{-1} of *S. aureus* JE2 WT and mutants defective for the sensor kinase components of TCS after 16 h incubation in human serum. Data represent the geometric mean ± geometric standard deviation of three independent experiments. Data were analysed by one-way ANOVA and no statistically significant differences were observed demonstrating there were no differences in survival in serum between the WT and any of the mutants. Source data are provided as a Source Data file.

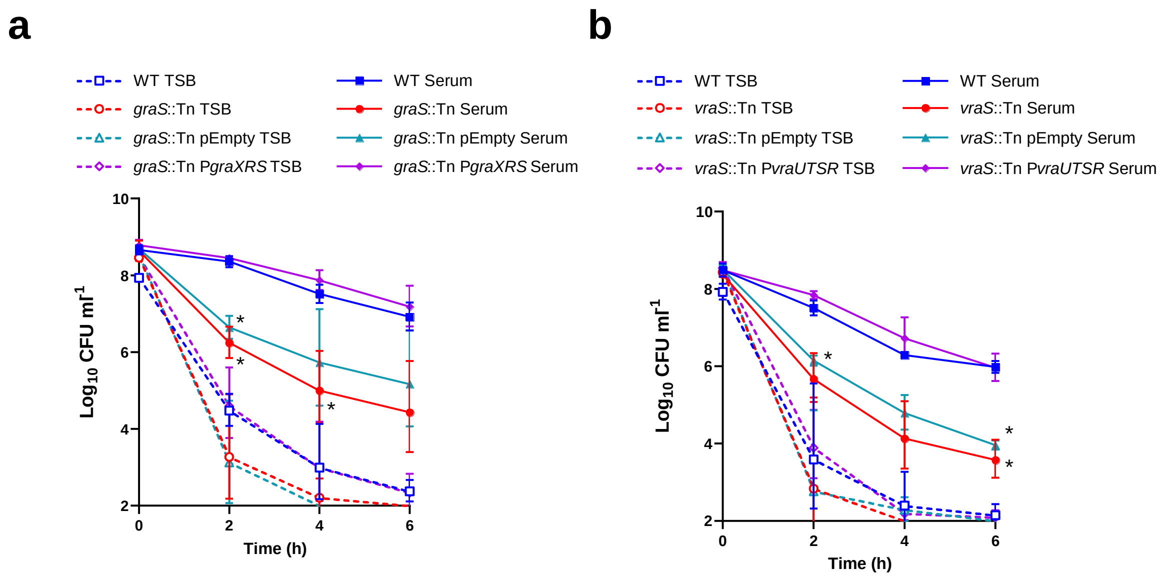

Supplementary Fig. 5. Complementation of the *vraS*::Tn and *graS*::Tn mutants restores tolerance to WT levels. Log$_{10}$ CFU ml^{-1} of TSB-grown and serum-adapted cultures of *S. aureus* JE2 WT, *graS*::Tn and *graS*::Tn complemented with pCN34 or P*graXRS* **(a)** and WT, *vraS*::Tn and *vraS*::Tn complemented with empty pCN34 or P*vraUTSR* **(b)** over 6 h incubation in serum with 80 µg ml^{-1} daptomycin. Graphs represent the geometric mean ± geometric standard deviation of three independent experiments. * for **a**, P = 0.0008 (2 h *graS*::Tn pEmpty Serum), 0.0019 (2 h *graS*::Tn Serum), 0.0279 (4 h *graS*::Tn Serum). For **b**, P = 0.002 (2 h *vraS*::Tn pEmpty Serum), 0.0002 (6 h *vraS*::Tn pEmpty Serum), 0.0198 (6 h *vraS*::Tn Serum) determined by two-way ANOVA with Dunnett's *post-hoc* test, serum-adapted WT compared to serum-adapted mutants at each time-point. Some data points fell below the limit of detection of 100 CFU ml^{-1}. Source data are provided as a Source Data file.

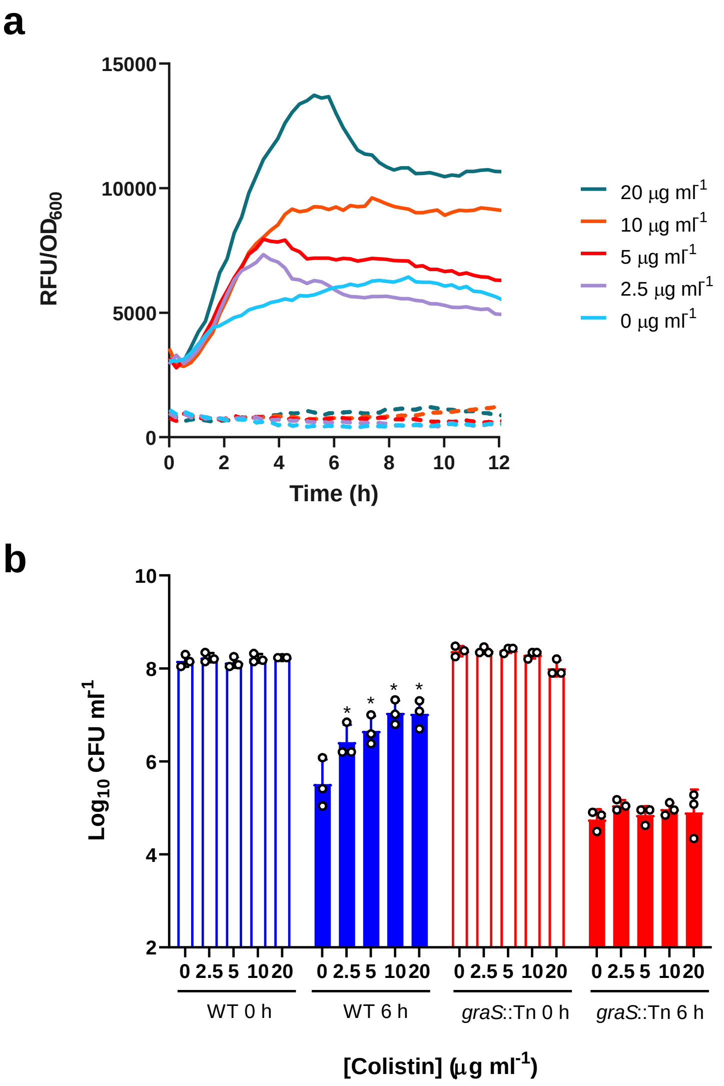

Supplementary Fig. 6. Colistin activates GraRS signalling and triggers daptomycin tolerance. TSB-grown cultures of *S. aureus* JE2 WT (solid lines) and the *graS*::Tn mutant (dashed lines) containing P*dltA*-*gfp* were exposed to various concentrations of colistin (2.5 – 20 µg ml^{-1}) in RPMI 1640 (**a**). GFP fluorescence (RFU) and OD_{600} were measured every 15 min for 12 h. Fluorescence values were divided by OD_{600} measurements to normalise for changes in cell density. *S. aureus* JE2 WT and *graS*::Tn mutant were each incubated for 16 h in RPMI 1640 only or RPMI 1640 supplemented with indicated concentrations of colistin before determination of CFU counts prior to (0 h) and after (6 h) exposure to 80 µg ml^{-1} daptomycin (**b**). Graph in **a** represents the mean of three independent experiments and error bars have been omitted for clarity. Graph in **b** represents the geometric mean ± geometric standard deviation of three independent experiments. *, P = 0.0001 (2.5 µg ml^{-1}), < 0.0001 (5 µg ml^{-1}), < 0.0001 (10 µg ml^{-1}), < 0.0001 (20 µg ml^{-1}) determined by two-way ANOVA with Dunnett's *post-hoc* test, RPMI 1640 and colistin compared to RPMI 1640 only. RFU, relative fluorescent units; OD_{600}, optical density at 600 nm. Source data are provided as a Source Data file.

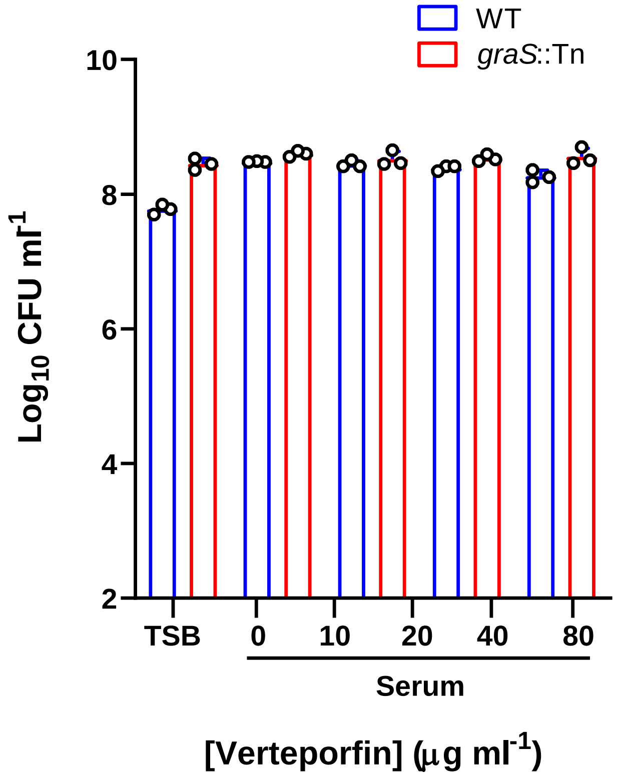

Supplementary Fig. 7. Exposure of *S. aureus* to verteporfin does not affect bacterial survival in serum. Log_{10} CFU ml^{-1} of *S. aureus* JE2 WT grown in TSB or after 16 h incubation in human serum with indicated concentrations of verteporfin. Data represent the geometric mean ± geometric standard deviation of three independent experiments. Data were analysed by two-way ANOVA and no statistically significant differences were observed between bacteria incubated in serum ± verteporfin confirming that verteporfin does not compromise *S. aureus* survival in serum in the absence of daptomycin. Source data are provided as a Source Data file.

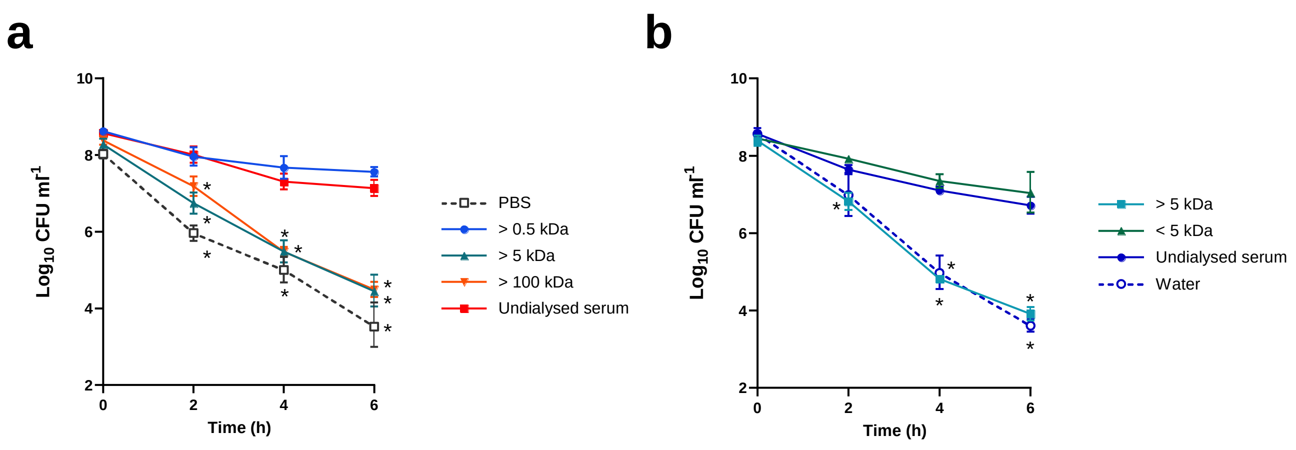

Supplementary Fig. 8. A 0.5 – 5 kDa fraction of serum is necessary and sufficient to trigger daptomycin tolerance. Serum was fractionated by dialysis against PBS using membranes with various molecular weight cut-offs. Next, CFU ml^{-1} were determined for *S. aureus* JE2 WT which had been incubated for 16 h in serum fractions, or in PBS only or unfractionated serum, then exposed to 80 µg ml^{-1} daptomycin in fresh human serum over 6 h (**a**). Log_{10} CFU ml^{-1} of *S. aureus* JE2 WT which had been incubated for 16 h in < 5 kDa or > 5 kDa serum fractions, or water only or undialysed serum, then exposed to 80 µg ml^{-1} daptomycin in fresh human serum over 6 h (**b**). Graphs represent the geometric mean ± geometric standard deviation of three independent experiments and were analysed by two-way ANOVA with Dunnett's *post-hoc* test. (* for **a**, P = 0.0373 (>100 kDa 2 h), 0.0016 (>100 kDa 4 h), 0.0003 (>100 kDa 6 h), 0.0106 (>5 kDa 2 h), 0.0037 (>5 kDa 4 h), 0.0053 (>5 kDa 6 h), 0.0008 (PBS 2 h), 0.0033 (PBS 4 h), 0.0121 (PBS 6 h). For **b**, P = 0.0233 (>5 kDa 2 h), 0.0009 (>5 kDa 4 h), 0.0002 (>5 kDa 6 h), 0.0206 (Water 4 h), 0.0002 (Water 6 h) comparing undialysed serum vs serum fractions/PBS/water). Source data are provided as a Source Data file.

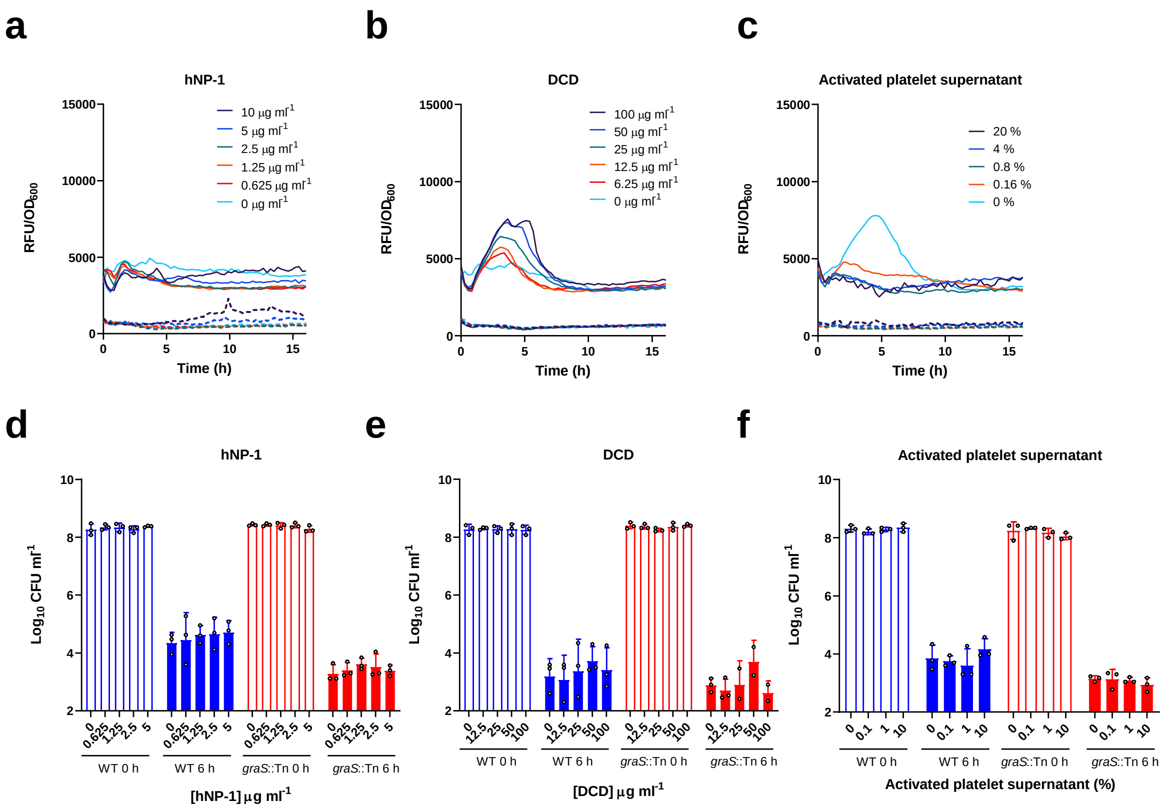

Supplementary Fig. 9. Human neutrophil peptide-1, dermcidin and platelet AMPs do not activate GraRS signalling or induce daptomycin tolerance. TSB-grown cultures of *S. aureus* JE2 WT (solid lines) and the *graS*::Tn mutant (dashed lines) containing P*dltA-gfp* were exposed to a range of concentrations of hNP-1 (**a**), DCD (**b**) or activated platelet supernatant (**c**) in RPMI 1640 and GFP fluorescence (RFU) and OD_{600} were measured every 15 min for 16 h. Fluorescence values were divided by OD_{600} measurements to normalise for changes in cell density. *S. aureus* JE2 WT and *graS*::Tn mutant were pre-incubated in RPMI 1640 containing hNP-1 (**d**), DCD (**e**) or activated platelet supernatant (**f**) for 16 h before CFU ml^{-1} were determine before (0 h) and after (6 h) exposure to 80 µg ml^{-1} daptomycin. Graphs in **a** – **c** represent the mean of at least two independent experiments with error bars omitted for clarity. Graphs in **d** – **f** represent the geometric mean ± geometric standard deviation of three independent experiments. Data in **d** – **f** were analysed by two-way ANOVA. No statistically significant differences were observed (RPMI 1640 + AMPs compared to RPMI 1640 alone). Source data are provided as a Source Data file.

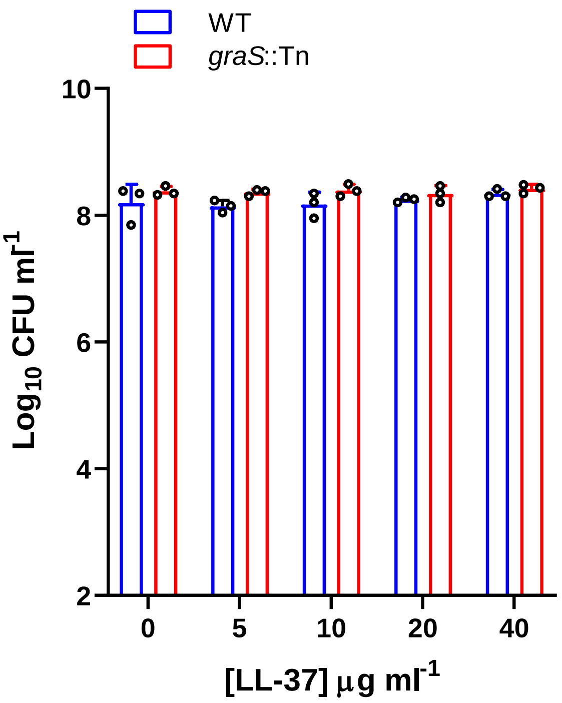

Supplementary Fig. 10. Exposure of *S. aureus* to LL-37 does not affect bacterial survival. Log_{10} CFU ml^{-1} of *S. aureus* JE2 WT after 16 h incubation in RPMI 1640 with indicated concentrations of LL-37. Data represent the geometric mean ± geometric standard deviation of three independent experiments. Data were analysed by two-way ANOVA and no statistically significant differences were observed (RPMI 1640 + LL-37 compared to RPMI 1640 alone). Source data are provided as a Source Data file.

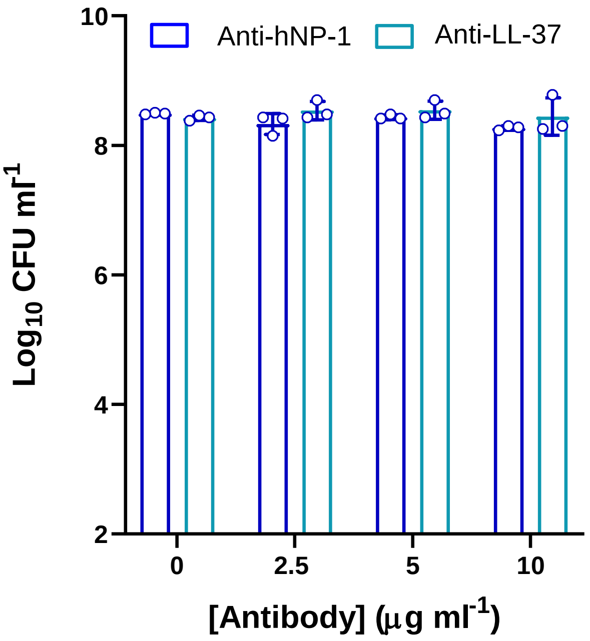

Supplementary Fig. 11. Exposure of *S. aureus* to anti-hNP-1 or anti-LL-37 antibodies did not affect bacterial survival. Log_{10} CFU ml^{-1} of *S. aureus* JE2 WT after incubation for 16 h in human serum supplemented with indicated concentrations of antibodies against hNP-1 or LL-37. Data represent the geometric mean ± geometric standard deviation of three independent experiments. Data were analysed by two-way ANOVA and no statistically significant differences were observed. Source data are provided as a Source Data file.

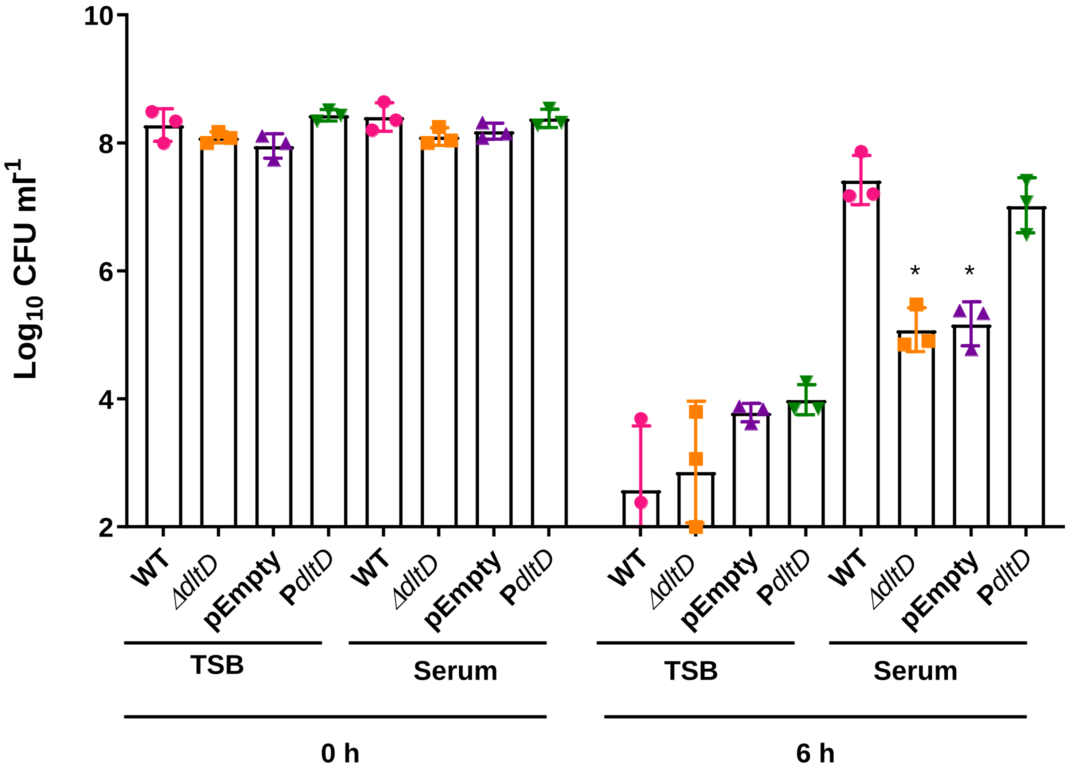

Supplementary Fig. 12. Complementation of *ΔdltD* mutant with WT *dltD* restores tolerance to WT levels. Log_{10} CFU ml^{-1} of TSB-grown and serum-adapted cultures of WT, the *ΔdltD* mutant or *ΔdltD* mutant complemented with either pCN34 or P*dltD* after a 16 h incubation in serum (0 h) and after exposure to daptomycin in serum (6 h). Graph represents the geometric mean ± geometric standard deviation of three independent experiments (one data point fell below the limit of detection of 100 CFU ml^{-1}). Data were analysed by two-way ANOVA with Dunnett's *post-hoc* test (* P < 0.0001 (Δ*dltD* serum 6 h), < 0.0001 (pEmpty serum 6 h) comparing serum-adapted WT with serum-adapted mutants). Source data are provided as a Source Data file.

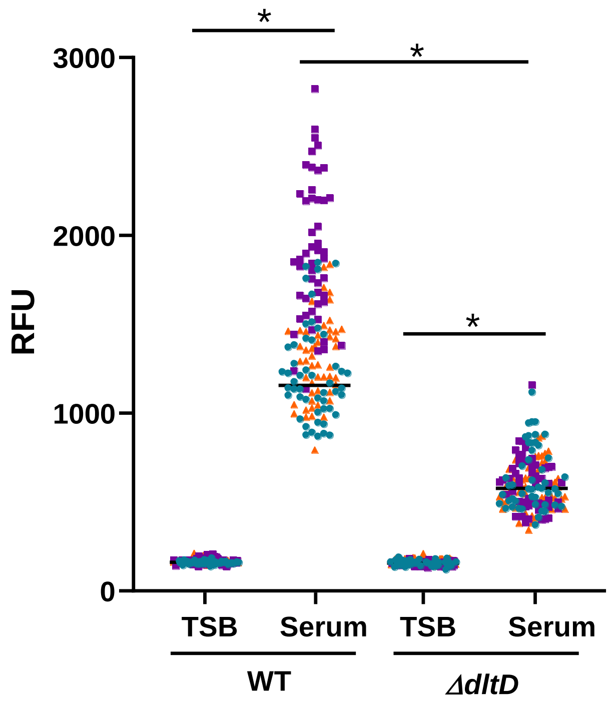

Supplementary Fig. 13. The *ΔdltD* mutant incorporates less HADA than WT during serum adaptation. The fluorescence of individual TSB-grown and serum-adapted WT and *ΔdltD* cells was quantified. Graph represents the fluorescence of 50 cells per biological replicate (150 cells in total) with the mean of the three replicates indicated. Each biological replicate is depicted in a different colour. Data were analysed by Kruskal Wallis test (* P = < 0.0001 (WT TSB vs Serum), < 0.0001 (WT Serum vs Δ*dltD* Serum), < 0.0001 (Δ*dltD* TSB vs Δ*dltD* Serum)). Source data are provided as a Source Data file.

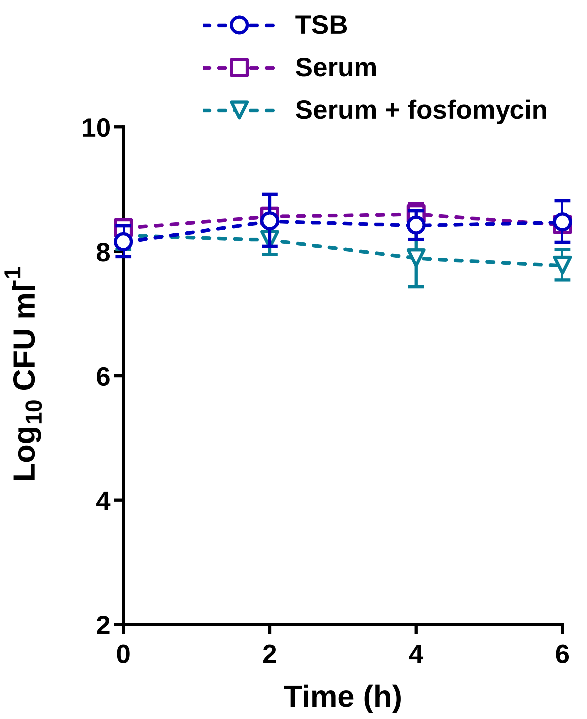

Supplementary Fig. 14. Inhibition of peptidoglycan synthesis during serum adaptation did not affect bacterial viability. Log_{10} CFU ml^{-1} over 6 h of TSB-grown *S. aureus* or cultures which had been incubated in serum supplemented, or not, with 64 µg ml^{-1} fosfomycin for 16 h. Graph represents the geometric mean ± geometric standard deviation of three independent experiments. No statistically significant differences were observed (two-way ANOVA, log_{10} CFU ml^{-1} at each time-point vs 0 h). Source data are provided as a Source Data file.

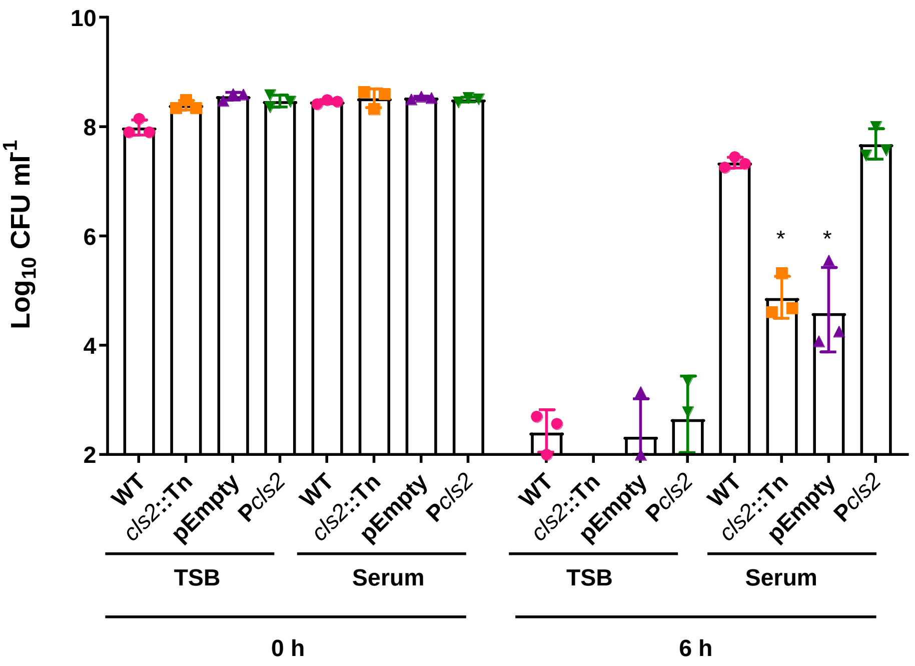

Supplementary Fig. 15. Complementation of the *cls2*::Tn mutant restores daptomycin tolerance. Log$_{10}$ CFU ml^{-1} of TSB-grown and serum-adapted cultures of *S. aureus* JE2 WT, *cls2*::Tn or *cls2*::Tn complemented with empty pCN34 or P*cls2* before (0 h) or after (6 h) exposure to 80 µg ml^{-1} daptomycin in serum. Graph represents the geometric mean ± geometric standard deviation of three independent experiments (some data points fell below the limit of detection of 100 CFU ml^{-1}). Data were analysed by two-way ANOVA with Dunnett's *post-hoc* test (* $P < 0.0001$ (*cls*2::Tn serum 6 h), < 0.0001 (pEmpty serum 6 h) comparing serum-adapted WT with serum-adapted mutants). Source data are provided as a Source Data file.
