# Supplementary material for: Human serum triggers antibiotic tolerance in Staphylococcus aureus
Source: Nat Commun. 2022 Apr 19;13:2041. doi: 10.1038/s41467-022-29717-3 (PMC9018823; doi:10.1038/s41467-022-29717-3)
Supplement: Supplementary file 2 — Reporting Summary [file 41467_2022_29717_MOESM2_ESM.pdf]

nature portfolio

Corresponding author(s): Andrew Edwards

Last updated by author(s): Mar 7, 2022

Reporting Summary

Nature Portfolio wishes to improve the reproducibility of the work that we publish. This form provides structure for consistency and transparency in reporting. For further information on Nature Portfolio policies, see our Editorial Policies and the Editorial Policy Checklist.

Statistics

For all statistical analyses, confirm that the following items are present in the figure legend, table legend, main text, or Methods section.

n/a	Confirmed	
☐	☒	The exact sample size (*n*) for each experimental group/condition, given as a discrete number and unit of measurement
☐	☒	A statement on whether measurements were taken from distinct samples or whether the same sample was measured repeatedly
☐	☒	The statistical test(s) used AND whether they are one- or two-sided<br>*Only common tests should be described solely by name; describe more complex techniques in the Methods section.*
☐	☒	A description of all covariates tested
☐	☒	A description of any assumptions or corrections, such as tests of normality and adjustment for multiple comparisons
☐	☒	A full description of the statistical parameters including central tendency (e.g. means) or other basic estimates (e.g. regression coefficient) AND variation (e.g. standard deviation) or associated estimates of uncertainty (e.g. confidence intervals)
☐	☒	For null hypothesis testing, the test statistic (e.g. *F*, *t*, *r*) with confidence intervals, effect sizes, degrees of freedom and *P* value noted<br>*Give P values as exact values whenever suitable.*
☒	☐	For Bayesian analysis, information on the choice of priors and Markov chain Monte Carlo settings
☒	☐	For hierarchical and complex designs, identification of the appropriate level for tests and full reporting of outcomes
☒	☐	Estimates of effect sizes (e.g. Cohen's *d*, Pearson's *r*), indicating how they were calculated

Our web collection on statistics for biologists contains articles on many of the points above.

Software and code

Policy information about availability of computer code

Data collection	Zen version 2.5 (Blue edition)
Data analysis	Graphpad Prism version 8.0, ImageJ 1.52a, Zen 2.5 (Blue edition)

For manuscripts utilizing custom algorithms or software that are central to the research but not yet described in published literature, software must be made available to editors and reviewers. We strongly encourage code deposition in a community repository (e.g. GitHub). See the Nature Portfolio guidelines for submitting code & software for further information.

Data

Policy information about availability of data

All manuscripts must include a data availability statement. This statement should provide the following information, where applicable:

- Accession codes, unique identifiers, or web links for publicly available datasets
- A description of any restrictions on data availability
- For clinical datasets or third party data, please ensure that the statement adheres to our policy

The datasets generated during the current study are available included in this published article (and its supplementary information files).

Field-specific reporting

Life sciences study design

All studies must disclose on these points even when the disclosure is negative.

Sample size	No method was used to predetermine sample size. Consistent with similar studies to those described here, all assays used 2-5 independent replicates as detailed in the figure legends, with replicate number based on the necessary sample size to test for significant differences between conditions, mutants or other variables.
Data exclusions	No data were excluded.
Replication	All replicates are included from 2-5 independent experiments, shown as individual data points where possible. All experiments were successfully completed.
Randomization	No experiments were performed on humans or animals and therefore randomisation is not applicable.
Blinding	No blinding was used as no subjective measurements were made.

Reporting for specific materials, systems and methods

We require information from authors about some types of materials, experimental systems and methods used in many studies. Here, indicate whether each material, system or method listed is relevant to your study. If you are not sure if a list item applies to your research, read the appropriate section before selecting a response.

Materials & experimental systems

n/a	Involved in the study	
☐	☒	Antibodies
☒	☐	Eukaryotic cell lines
☒	☐	Palaeontology and archaeology
☒	☐	Animals and other organisms
☐	☒	Human research participants
☒	☐	Clinical data
☒	☐	Dual use research of concern

Methods

n/a	Involved in the study	
☒	☐	ChIP-seq
☒	☐	Flow cytometry
☒	☐	MRI-based neuroimaging

Antibodies

Antibodies used	Polyclonal sheep IgG anti-LL-37 (AF7497, Lot CGMD0220091), Polyclonal sheep IgG anti-hNP-1 (AF8198, Lot CHWR0118031) (both R&D Systems).
Validation	Anti- LL-37 demonstrated to be functional in western immunoblotting, resulting in a single band of the expected mass from a lysate of human peripheral blood mononuclearcytes.<br>Anti-hNP-1 demonstrated to be functional in immunohistochemistry of human small intestinal section and ELISA for human alpha-defensin.

Human research participants

Policy information about studies involving human research participants

Population characteristics	Blood was used from healthy male (2) and female (1) donors aged between 25 and 35 years.
Recruitment	Advertisement for healthy donors. Donors were not rewarded and were randomly selected from those who responded to the advertisement. Therefore, we do not anticipate any impact from self-selection or other biases.
Ethics oversight	Ethical approval for drawing and processing human blood was obtained from the Regional Ethics Committee of Imperial College healthcare tissue bank (Imperial College London) and the Imperial NHS Trust Tissue Bank (REC Wales approval no. 12/WA/0196 and ICHTB HTA license no. 12275). Written informed consent was obtained from the donors prior to taking samples.

Note that full information on the approval of the study protocol must also be provided in the manuscript.
