# Supplementary figures and images for: Human serum triggers antibiotic tolerance in Staphylococcus aureus
Source: Nat Commun. 2022 Apr 19;13:2041. doi: 10.1038/s41467-022-29717-3 (PMC9018823; doi:10.1038/s41467-022-29717-3)

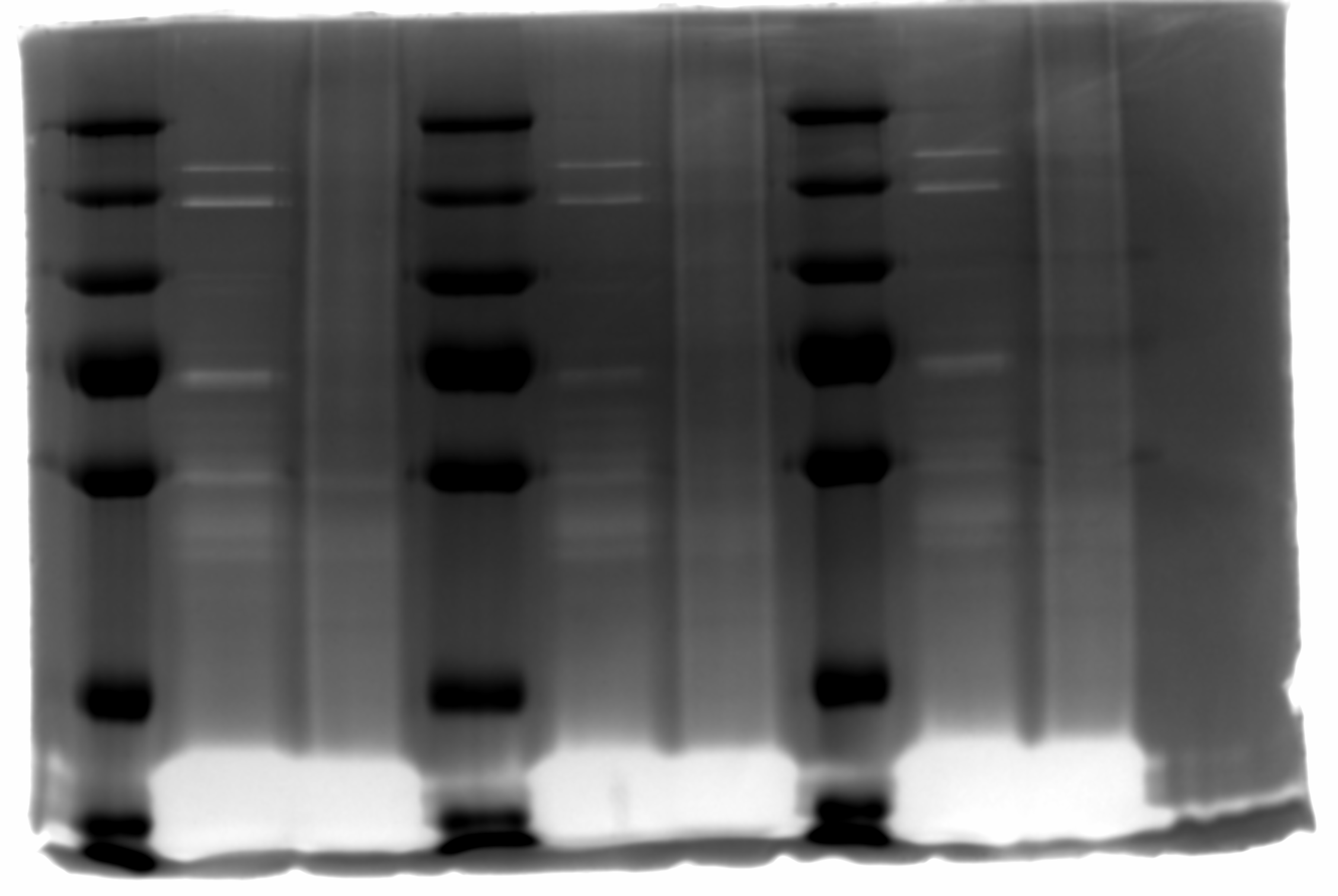

Supplement: Supplementary file 4 — Source data file [file 41467_2022_29717_MOESM4_ESM.zip › Fig. 6B uncropped.tif]

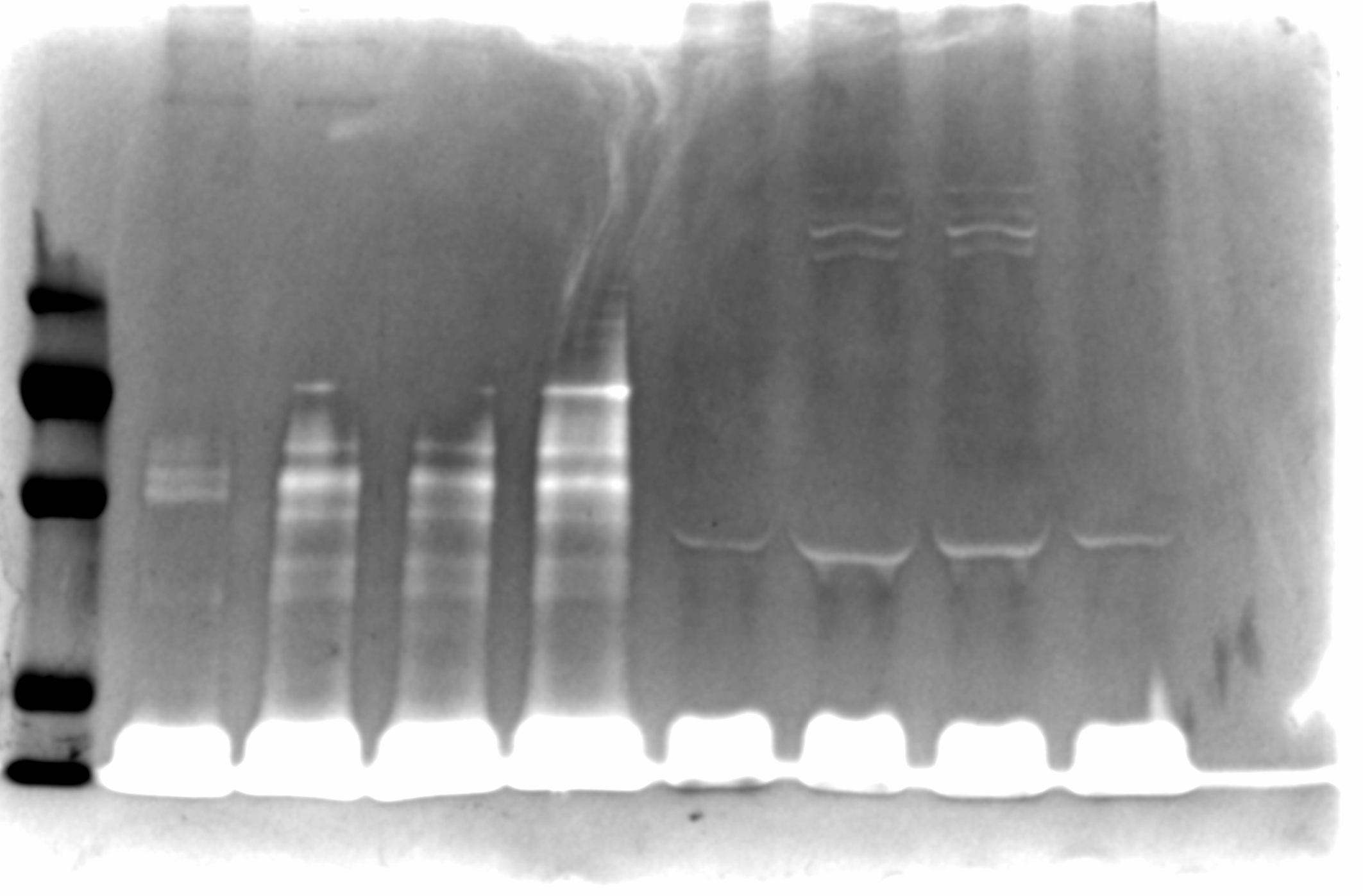

Supplement: Supplementary file 4 — Source data file [file 41467_2022_29717_MOESM4_ESM.zip › Fig. 6D uncropped.tif]
